# Pediatric melanoma in Arizona: Demographic, tumor, and treatment characteristics from the Arizona cancer registry

**DOI:** 10.1016/j.jdin.2026.01.008

**Published:** 2026-02-02

**Authors:** Jake Besch, Jenna E. Koblinski, Mehrdad Motamed, Claire Yee, Harper Price, Collin M. Costello

**Affiliations:** a Department of Dermatology, Mayo Clinic, Scottsdale, Arizona; b Department of Dermatology, Emory University, Atlanta, Georgia; c Department of Quantitative Health Science Research, Mayo Clinic, Scottsdale, Arizona; d Department of Dermatology, Phoenix Children’s Hospital, Phoenix, Arizona

**Keywords:** Arizona Cancer Registry, melanoma, pediatric dermatology, pediatric melanoma, skin cancer

*To the Editor:* Pediatric melanoma is relatively understudied compared to its adult counterpart, though recent studies have provided valuable insights.^1^ A 2013 study showed that between 1973 and 2009, incidence increased by an average of 2% per year.^2^ While more recent studies have shown that the incidence may be decreasing, additional research is necessary, particularly to examine pediatric melanoma at a state level.^3^ Arizona offers a unique opportunity to investigate pediatric melanoma, given its high level of UV exposure and 1 of the highest pediatric melanoma mortality rates nationally.^4^

Our study aimed to examine the demographic, staging, and treatment data for pediatric patients diagnosed with melanoma in Arizona, and to analyze associations with age, race, and sex. Records of patients under 18 diagnosed with melanoma in Arizona (2000-2021) were obtained from the Arizona Cancer Registry. See Supplementary Methods, available via Mendeley at https://data.mendeley.com/datasets/bdg5yj4jzf/1.

A total of 141 patients were included (Table I). The average age at diagnosis was 13.1 years (standard deviation = 4.63). A total of 72.4% were between 13 and 17 years old. Most patients were female (56.7%), white (93.6%), non-Hispanic (78.0%), with the head/neck being the most common region involved (29.8%). Most cases were in situ (11.1%) or localized (66.7%). Average Breslow thickness for invasive melanomas was 1.7 mm (standard deviation = 1.6). A total of 55.9% of patients had regional node biopsy and 14.9% were positive. One hundred and thirty-one patients (92.9%) had excision, and no residual tumor was noted in 120 (86.3%) cases. Thirteen (13.8%) patients received systemic therapy before or after surgery. Eleven (7.8%) patients received immunotherapy as part of the first course of treatment, while 6 (4.2%) received chemotherapy. A total of 94.3% of patients were alive at the last follow-up, and 82.3% had no evidence of tumor.

**Table I:** Demographic, tumor, staging, and treatment data for pediatric melanoma patients

Category	Total (*N* = 141)
Race, *n* (%)	
White	132 (93.6%)
American Indian or Alaska Native	3 (2.1%)
Other	1 (0.7%)
Unknown	5 (3.5%)
Hispanic origin, *n* (%)	
Non-Hispanic/Latino	110 (78.0%)
Mexican	4 (2.8%)
Hispanic NOS	19 (13.5%)
Unknown	8 (5.7%)
Age group, *n* (%)	
0-8 y	26 (18.4%)
9-12 y	13 (9.2%)
13-17 y	102 (72.3%)
Age at diagnosis	
*N*	141
Mean (SD)	13.1 (4.63)
Median (IQR)	15.0 (12.0, 17.0)
Range	0.0, 17.0
Sex, *n* (%)	
Male	61 (43.3%)
Female	80 (56.7%)
Stage, *n* (%)	
In situ	13 (11.1%)
Localized	78 (66.7%)
Regional	15 (12.8%)
Unstaged/unknown	11 (9.4%)
Missing	24
Regional node biopsied, *n* (%)	
None	60 (42.6%)
Sentinel/other regional biopsy	79 (55.9%)
Unknown or NA	2 (1.4%)
Regional nodes positive, *n* (%)	
All negative	57 (40.4%)
1+ nodes positive	21 (14.9%)
None examined	57 (40.4%)
Unknown	6 (4.3%)
Breslow tumor thickness (mm)	
*N*	21
Mean (SD)	1.7 (1.57)
Median (IQR)	1.1 (0.6, 1.7)
Range	0.5, 6.0
Missing	120
Ulceration, *n* (%)	
Not identified	20 (83.3%)
Present	2 (8.3%)
Unknown	2 (8.3%)
Missing	117
Metastasis at diagnosis, *n* (%)	
None	67 (93.1%)
Metastasis	1 (1.4%)
Unknown	4 (5.6%)
Missing	69
Primary site, *n* (%)	
Head and neck	42 (29.8%)
Trunk	41 (29.1%)
Upper limb	29 (20.6%)
Lower limb	27 (19.1%)
Skin, NOS	2 (1.4%)
Histologic type ICD-O-3, *n* (%)	
MIS or MM NOS	108 (76.6%)
Nodular	4 (2.8%)
Superficial spreading	18 (12.8%)
Other	2 (1.4%)
MM in giant pigmented nevus	4 (2.8%)
Epithel. and spindle	5 (3.5%)
Primary site surgery, *n* (%)	
None	4 (2.8%)
Excision	131 (93%)
Mohs	4 (2.8%)
Surgery, NOS	2 (1.4%)
Surgical margins, *n* (%)	
No residual	120 (86.3%)
Residual	5 (3.6%)
Not evaluable	3 (2.2%)
No surgery	4 (2.9%)
Unknown	7 (5.0%)
Missing	2
Radiation, *n* (%)	
None	139 (98.6%)
Radiation	1 (0.7%)
Unknown	1 (0.7%)
Chemotherapy, *n* (%)	
None	134 (95.0%)
Chemotherapy	6 (4.2%)
Unknown	1 (0.7%)
Immunotherapy, *n* (%)	
None	129 (91.5%)
As first course	11 (7.8%)
Unknown	1 (0.7%)
Vital status, *n* (%)	
Dead	8 (5.7%)
Alive	133 (94.3%)
Cancer status, *n* (%)	
No tumor	116 (82.3%)
Tumor	14 (9.9%)
Unknown	11 (7.8%)

*Epithel. & spindle, Combined*, Mixed epithelioid & spindle cell melanoma, Epithelioid cell melanoma, and Spindle cell melanoma; *ICD-O-3*, International Classification of diseases for oncology, 3rd edition; *IQR*, interquartile range; *MIS*, melanoma in situ; *MM*, malignant melanoma; *n*, number; *N,* total population; *NOS*, not otherwise specified; *nNSD*, standard deviation; Stage, Combined SEER Summary stage 1977, 2000, and 2018.

No significant differences (*P* > .05) in Breslow thickness, ulceration, likelihood of undergoing regional node biopsy, node positivity, receiving immunotherapy, cancer status, or vital status were observed based on age, race, or sex (Table II). As age increased, the odds of being diagnosed with in situ (*P* = .0153) or localized (*P* = .0098) disease compared to regional disease increased. Age was significantly associated (*P* = .0148) with likelihood of receiving chemotherapy. Females were more likely to be diagnosed with melanoma on the lower extremities (*P* = .0435), while males were more likely to be diagnosed on the head and neck (*P* = .0435).

**Table II:** Univariate analysis: Effect of race, age, and sex on pediatric melanoma outcomes

Outcome	Predictor	Odds ratio (95% CI)	*P* value
Breslow thickness	Race	NA	*P* = .9521
	Age	NA	*P* = .1322
	Sex	NA	*P* = .6900
Ulceration	Race	NA	*P* = .9719
	Age	1.153 (0.902-1.472)	*P* = .2555
	Sex	1.500 (0.082-27.606)	*P* = .7849
Lymph node biopsy	Race	1.086 (0.279-4.229)	*P* = .9051
	Age	1.074 (0.993-1.162)	*P* = .0646
	Sex	1.005 (0.512-1.972)	*P* = .9883
Regional node positive	Race	0.722 (0.173-3.014)	*P* = .6553
	Age	0.995 (0.925-1.070)	*P* = .8925
	Sex	1.042 (0.528-2.053)	*P* = .9061
Chemotherapy	Race	NA	*P* = .9777
	Age	1.195 (1.035-1.379)	***P* = .0148**
	Sex	1.526 (0.270-8.622)	*P* = .6322
Immunotherapy	Race	NA	*P* = .7095
	Age	1.010 (0.881-1.158)	*P* = .8890
	Sex	NA	*P* = .8562
Stage (regional as reference)			
In situ	Race	1.167 (0.066-20.722)	*P* = .9164
Localized		0.757 (0.079-7.285)	*P* = .8094
Unstaged		1.400 (0.078-25.144)	*P* = .8194
In situ	Age	1.324 (1.055-1.661)	***P* = .0153**
Localized		1.146 (1.033-1.272)	***P* = .0098**
Unstaged		1.075 (0.931-1.241)	*P* = .3248
In situ	Sex	0.714 (0.158-3.231)	*P* = .6621
Localized		0.930 (0.307-2.818)	*P* = .8982
Unstaged		1.371 (0.288-6.534)	*P* = .6917
Site (head and neck as reference)			
LE	Race	NA	*P* = .9873
Trunk		0.487 (0.084-2.818)	*P* = .4220
UE		1.096 (0.226-5.310)	*P* = .9092
LE	Age	0.969 (0.880-1.066)	*P* = .5157
Trunk		1.046 (0.949-1.153)	*P* = .3666
UE		1.059 (0.947-1.184)	*P* = .3151
LE	Sex	0.348 (0.125-0.970)	***P* = .0435**
Trunk		0.647 (0.272-1.537)	*P* = .3235
UE		0.505 (0.192-1.325)	*P* = .1652
Cancer status (no tumor as reference)	Race	1.038 (0.120-8.978)	*P* = .9726
	Age	0.973 (0.870-1.089)	*P* = .6378
	Sex	0.956 (0.312-2.930)	*P* = .9371
Vital status	Race	NA	*P* = .9744
	Age	0.970 (0.819-1.149)	*P* = .7245
	Sex	0.750 (0.180-3.127)	*P* = .6929

Bolded *P* values demonstrate statistically significant *P* values, defined as less than 0.05.

*H&N*, Head and neck; *LE*, lower extremities; *Stage*, Combined SEER Summary stage 1977, 2000, and 2018; *UE*, upper extremities.

Our results show that older patients were more likely to be diagnosed with in situ or localized disease, yet were also more likely to receive chemotherapy. Consistent with prior studies, males were more likely to be diagnosed on the head and neck, while females were more likely to be diagnosed on the lower extremities.^5^ Limitations include inclusion of historical diagnostic and treatment practices, inability to stratify by pubertal status or consistently capture biologically distinct subtypes, and registry grouping that may include Spitz-lineage tumors within the melanoma NOS category. Although multiple variables had a low sample size, this study captures all reported cases of pediatric melanoma in the state of Arizona, representing an important step toward exploring melanoma trends in high-risk locations. Further studies are needed to explore the clinical and sociodemographic factors influencing treatment decisions and outcomes in pediatric melanoma.

### Declaration of generative AI and AI-assisted technologies in the writing process

Not applicable.

## Conflicts of interest

None disclosed. Unrelated to this study, Dr Costello was an investigator for Merck within the last 12 months and an investigator for DeepX Health more than 24 months ago.
